# Evolution of the bamboos (Bambusoideae; Poaceae): a full plastome phylogenomic analysis

**DOI:** 10.1186/s12862-015-0321-5

**Published:** 2015-03-18

**Authors:** William P Wysocki, Lynn G Clark, Lakshmi Attigala, Eduardo Ruiz-Sanchez, Melvin R Duvall

**Affiliations:** Biological Sciences, Northern Illinois University, 1425 W Lincoln Hwy, DeKalb, 60115-2861 IL USA; Ecology, Evolution and Organismal Biology, Iowa State University, 251 Bessey Hall, Ames, 50011-1020 IA USA; Instituto de Ecología AC, Centro Regional del Bajío, Biodiversidad y Sistemática, Av. Lázaro Cárdenas 253, Pátzcuaro, 61600 Michoacán Mexico

**Keywords:** Poaceae, Bambusoideae, Bamboos, Phylogenomics, Plastome, Organellar HGT, Chloroplast genome

## Abstract

**Background:**

Bambusoideae (Poaceae) comprise three distinct and well-supported lineages: tropical woody bamboos (Bambuseae), temperate woody bamboos (Arundinarieae) and herbaceous bamboos (Olyreae). Phylogenetic studies using chloroplast markers have generally supported a sister relationship between Bambuseae and Olyreae. This suggests either at least two origins of the woody bamboo syndrome in this subfamily or its loss in Olyreae.

**Results:**

Here a full chloroplast genome (plastome) phylogenomic study is presented using the coding and noncoding regions of 13 complete plastomes from the Bambuseae, eight from Olyreae and 10 from Arundinarieae. Trees generated using full plastome sequences support the previously recovered monophyletic relationship between Bambuseae and Olyreae. In addition to these relationships, several unique plastome features are uncovered including the first mitogenome-to-plastome horizontal gene transfer observed in monocots.

**Conclusions:**

Phylogenomic agreement with previous published phylogenies reinforces the validity of these studies. Additionally, this study presents the first published plastomes from Neotropical woody bamboos and the first full plastome phylogenomic study performed within the herbaceous bamboos. Although the phylogenomic tree presented in this study is largely robust, additional studies using nuclear genes support monophyly in woody bamboos as well as hybridization among previous woody bamboo lineages. The evolutionary history of the Bambusoideae could be further clarified using transcriptomic techniques to increase sampling among nuclear orthologues and investigate the molecular genetics underlying the development of woody and floral tissues.

**Electronic supplementary material:**

The online version of this article (doi:10.1186/s12862-015-0321-5) contains supplementary material, which is available to authorized users.

## Background

Bambusoideae are a lineage of perennial forest grasses (Poaceae) endemic to every continent except Europe and Antarctica [1,2]. The Bambusoideae comprise 115 genera and approximately 1450 species of bamboos [1]. Bambusoideae are divided into two morphologically distinct habits: woody and herbaceous bamboos. While woody bamboos display a wide range of morphological diversity, they do possess multiple shared characteristics. The woody bamboo syndrome includes strongly lignified culms, specialized culm leaves, complex vegetative branching, outer ligules on the foliage leaves, bisexual flowers, and gregarious monocarpy [1]. These bamboos, some of which can quickly grow up to 45 m in height, serve as an economically important source of building materials and other products for cultures in Central and South America, Asia, Africa, and Australia [3]. Their potential for rapid establishment combined with their extensive vegetative reproduction also make bamboos important ecologically as they can serve as forest habitats of their own and can affect the survival of sympatric woody species [4]. The gregarious, semelparous flowering patterns of woody bamboos and subsequent death of the parent plant can have ecological effects such as the increase in pest populations during the fruiting of *Melocanna baccifera* in regions of India [5] and the increase in eudicot sapling growth during the die-off of the dominant forest bamboo *Chusquea culeou* [6]. This pattern of flowering is correlated with increased generation times in this group, which in turn is correlated in bamboos and other grasses with shorter branch lengths in phylogenetic analyses [7] and fewer resolved nodes between certain closely related species.

Herbaceous bamboos are characterized by shorter and more weakly lignified shoots, less vegetative branching, unisexual flowers, and annual or seasonal flowering patterns [1]. The flowering phenology of herbaceous bamboos is correlated with an increase in the substitution rates observed in chloroplast loci. This has at least two consequences relevant to bambusoid plastome phylogenomics. First, phylogenetic resolution and support within this group are likely to be increased due to higher numbers of informative sites. At the same time, long branches are produced with the potential for long-branch attraction artifacts between herbaceous bamboos and non-bambusoid outgroups. Phylogenomic results can be more realistically interpreted taking these effects into account.

Molecular studies have placed Bambusoideae in the BEP (Bambusoideae, Ehrhartoideae, Pooideae) clade of Poaceae. A sister group relationship between Bambusoideae and Pooideae has been strongly supported [8,9] although morphological synapomorphies have yet to be found that unite these two subfamilies. Bambusoideae can be divided into three well-supported monophyletic tribes: the woody Arundinarieae and Bambuseae, and the herbaceous Olyreae [1].

The Bambuseae are native to tropical areas in both the Old and New World. This tribe comprises two clades that correspond to Old and New World species [2,10]. Phylogenetic studies that use plastid markers generally place Olyreae as the sister group to Bambuseae in well-supported trees [2,11,12]. Olyreae are exclusively distributed in the New World except for *Buergersiochloa bambusoides*, a species endemic to New Guinea, and *Olyra latifolia*, which is found widely distributed in Africa/Madagascar as well as in the New World [13]. However, the African origin of the *O. latifolia* population has been debated [14]. Like the Bambuseae, the Arundinarieae include woody bamboos found in both the Old and New World, with a basically Laurasian distribution pattern, but unlike most Bambuseae they are well-adapted to temperate environments.

Although paraphyly of the woody syndrome in Bambusoideae is well supported by tree analyses that use maternally inherited chloroplast phylogenetic markers [8], this has been a subject of debate. Network analyses have revealed that the phylogenetic placement of Olyreae is less certain than previously reported [2]. This is also because to be consistent with the chloroplast phylogeny, the woody bamboo syndrome would have either evolved twice independently (once in each of the ancestors of the Bambuseae and Arundinarieae) or arisen once in the common ancestor of the Bambusoideae and then subsequently been lost in the Olyreae. A hypothesized single origin of the woody bamboo syndrome, which has been most recently supported by Triplett et al. [15], is evolutionarily more parsimonious than these scenarios.

In the past three years, full plastome phylogenomic analyses have been used to address evolutionary problems in the Bambusoideae. These analyses have been variously applied in Bambusoideae to resolve subfamilial relationships [9,16,17] and investigate biogeographical patterns [12,18]. Full plastome analysis can also provide enough information to resolve difficult interspecific relationships. This is an issue that is especially relevant to woody bamboos, which generally hybridize readily and exhibit very long generation times [19,20]. While studies such as Kelchner et al. [2] and Triplett & Clark [21] have used selected chloroplast markers to infer maternally inherited evolutionary signal within Bambusoideae, our objective is to use all coding and non-coding regions within the chloroplast to increase the number of informative sites. Here, a full plastome phylogeny was generated using 13 tropical woody species, 10 temperate woody species and eight herbaceous species with 17 newly sequenced and 15 existing bambusoid plastomes plus two outgroup plastomes.

## Results

### Assembly and alignment of plastomes

Read and contig assembly yielded complete plastomes for 18 bamboos and one ehrhartoid grass. Plastome lengths ranged from 135,320—143,810 base pairs (bp). Lengths of each plastome region are reported in Table 1. A multi-plastome sequence alignment was 132,707 bp in length after excluding one of the major inverted repeat (IR) regions. Removal of alignment columns containing gaps reduced the alignment length to 97,593 bp. The sequence alignment containing only protein coding regions was 54,548 bp in length and 52,941 bp after removal of gapped positions. See Table 2 for more information on sequencing techniques and results.

**Table 1:** **NCBI nucleotide database accession numbers and lengths of regions and subregions for plastomes analyzed in this study**

**Taxon**	**Tribe**	**Total length**	**LSC** ^**a**^	**SSC** ^**b**^	**IR** ^**c**^	**Accession**	**Voucher**
*Acidosasa purpurea*	Arundinarieae	139,697	83,273	12,834	21,795	NC015820	N/A
*Arundinaria appalachiana*	Arundinarieae	139,547	83,222	12,717	21,804	NC023934	N/A
*Arundinaria gigantea*	Arundinarieae	138,935	82,632	12,709	21,797	NC020341	N/A
*Arundinaria tecta*	Arundinarieae	139,499	83,161	12,730	21,804	NC023935	N/A
*Ferrocalamus rimosivaginus*	Arundinarieae	139,467	83,091	12,718	21,829	NC015831	N/A
*Indocalamus longiauritus*	Arundinarieae	139,668	83,273	12,811	21,792	NC015803	N/A
*Phyllostachys edulis*	Arundinarieae	139,679	83,213	12,870	21,798	NC015817	N/A
*Phyllostachys nigra*	Arundinarieae	139,839	83,234	12,879	21,863	NC015826	N/A
*Phyllostachys propinqua*	Arundinarieae	139,704	83,228	12,878	21,799	NC016699	N/A
*Thamnocalamus spathiflorus*	Arundinarieae	139,498	83,310	12,594	21,797	KJ871005	LC 1319 (ISC)
*Bambusa arnhemica*	Bambuseae	139,287	82,790	12,901	21,798	KJ870989	PMP 1846 (CAN)
*Bambusa bambos*	Bambuseae	142,772	79,972	12,868	24,966	KJ870988	BI 1
*Bambusa emeiensis*	Bambuseae	139,491	82,976	12,911	21,802	NC015830	N/A
*Bambusa oldhamii*	Bambuseae	139,347	82,889	12,878	21,790	NC012927	N/A
*Chusquea liebmannii*	Bambuseae	138,001	81,501	12,892	21,804	KJ871001	LC & LA 1710 (ISC)
*Chusquea spectabilis*	Bambuseae	136,848	80,743	12,671	21,717	KJ870990	XL & LC 919 (ISC)
*Dendrocalamus latiflorus*	Bambuseae	139,369	82,975	12,884	21,755	NC013088	N/A
*Greslania* sp.	Bambuseae	139,264	82,581	12,979	21,852	KJ870993	GM (MO)
*Guadua weberbaueri*	Bambuseae	135,320	82,803	12,929	19,794	KP793062	XL & MK 582 (TULV)
*Hickelia madagascariensis*	Bambuseae	138,276	81,925	12,743	21,804	KJ870994	SD 1349 (K)
*Neololeba atra*	Bambuseae	139,395	82,905	12,926	21,782	KJ870996	LC & JT 1663 (ISC)
*Olmeca reflexa*	Bambuseae	136,213	82,726	12,945	20,271	KJ870997	Francisco Botanical Garden 312 (GCR)
*Otatea acuminata*	Bambuseae	136,351	82,859	12,948	20,272	KJ871003	LC & WZ 1348 (ISC)
*Buergersiochloa bambusoides*	Olyreae	138,122	81,746	12,856	21,760	KJ871000	SD 1365 (Kew)
*Cryptochloa strictiflora*	Olyreae	134,332	80,554	12,766	20,506	JX235348	N/A
*Diandrolyra* sp.	Olyreae	137,469	81,752	13,259	21,229	KJ870991	LC 1301 (ISC)
*Eremitis* sp.	Olyreae	143,810	80,984	13,232	24,797	KJ870992	LC & WZ 1343 (ISC)
*Lithachne pauciflora*	Olyreae	135,385	79,465	13,676	21,122	KJ871002	LC 1297 (ISC)
*Olyra latifolia*	Olyreae	135,834	80,642	12,770	21,211	KF515509	N/A
*Pariana radiciflora*	Olyreae	139,650	81,847	13,221	22,291	KJ871004	LC & WZ 1344 (ISC)
*Raddia brasiliensis*	Olyreae	135,739	80,713	13,000	21,013	KJ870998	LC & LA 1713 (ISC)
*Zizania aquatica*	Oryzeae (Ehrhartoideae)	136,354	82,009	12,587	20,879	KJ870999	JS 20870 (CAN)
*Lolium perenne*	Poeae (Pooideae)	135,246	80,000	12,428	21,409	NC009950	N/A

^a^Large Single Copy Region.

^b^Short Single Copy Region.

^c^Inverted Repeat Region.

LC, Lynn Clark; LA, Lakshmi Attigala; SD, Soejatmi Dransfield; WZ, Weiping Zhang; JS, Jeff Saarela; PMP, Paul Peterson; JT, Jimmy Triplett; BI, Bogor, Indonesia; GM, G. McPherson; MK, M. Kobayashi; XL, Ximena Londoño; GCR, Gilberto Cortés Rodríguez.

**Table 2:** **Sequencing details for all plastomes newly assembled for this study**

**Taxon**	**Tribe**	**Number of reads**	**Library preparation method**	**Sequencing method**	**Mean coverage**	**Number of scaffolded contigs**
*Thamnocalamus spathiflorus*	Arundinarieae	7,098,663	TruSeq	Single-end	54.8	5
*Bambusa arnhemica*	Bambuseae	2,292,120	TruSeq	Single-end	25.7	15
*Bambusa bambos*	Bambuseae	5,279,202	TruSeq	Single-end	53.2	3
*Chusquea liebmannii*	Bambuseae	23,707,569	TruSeq Nano	Paired-end	126.8	6
*Chusquea spectabilis*	Bambuseae	7,348,756	Nextera	Single-end	23.7	9
*Greslania* sp.	Bambuseae	13,881,568	Nextera	Single-end	142.1	3
*Guadua weberbaueri*	Bambuseae	29,431,971	Nextera	Single-end	94.9	9
*Hickelia madagascariensis*	Bambuseae	13,509,970	Nextera	Single-end	43.7	10
*Neololeba atra*	Bambuseae	28,569,106	TruSeq Nano	Paired-end	497.3	13
*Olmeca reflexa*	Bambuseae	5,400,472	Nextera	Single-end	51.3	6
*Otatea acuminata*	Bambuseae	14,532,488	TruSeq Nano	Paired-end	134.9	4
*Buergersiochloa bambusoides*	Olyreae	12,592,122	Nextera	Single-end	124.8	6
*Diandrolyra* sp.	Olyreae	10,004,619	Nextera	Single-end	100	4
*Eremitis* sp.	Olyreae	4,674,178	Nextera	Single-end	15.4	13
*Lithachne pauciflora*	Olyreae	14,773,417	Nextera	Single-end	233.1	4
*Pariana radiciflora*	Olyreae	23,398,974	TruSeq Nano	Paired-end	119.6	4
*Raddia brasiliensis*	Olyreae	6,828,240	Nextera	Single-end	40.1	3
*Zizania aquatica*	Oryzeae (Ehrhartoideae)	6,018,945	TruSeq	Single-end	66.3	3

### Unique plastome features

Plastomes are highly conserved chromosomes in which gene content, structure, and arrangement are quite similar across Poaceae [22]. When infrequent events such as large insertion/deletion (indel) mutations or inversions do occur, they take on greater significance because of their rarity and therefore higher chance of indicating shared ancestry. Four of these were observed here among bambusoid plastomes, in each case marking a single synapomorphic event in our phylogeny (see below).

**Figure 1:**
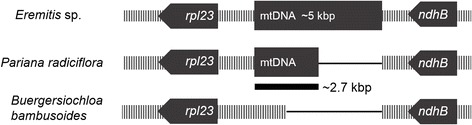
**Relative positions of putative mitochondrial insertions in the** ***Pariana radiciflora*** **and** ***Eremitis*** **sp. plastomes.** A diagram of the region in *Buergersiochloa bambusoides* is also included to illustrate an example of a typical grass plastome without the insertion. Solid bars represent relative gene positions, striped bars represent intergenic regions and thin lines represent gaps that were introduced to preserve downstream alignment. Note that this figure is not drawn to scale.

1) A 2,706 bp insertion exclusive to sampled members of the Parianinae (*Eremitis* sp*.* and *Pariana radiciflora*) was found in the *rpl23-ndhB* intergenic spacer of the *Pariana radiciflora* plastome, while the *Eremitis* sp*.* plastome possessed this insertion plus an additional 1,242 bp inserted on the 3′ end, giving the insertion a total length of 4,938 bp (Figure 1). A query of the NCBI nucleotide database using BLASTn [23] revealed the highest scoring hit to be mitochondrial sequence from *Ferrocalamus rimosivaginus*, a member of the Arundinarieae (98-99% nucleotide identity). Subsequent BLAST hits were all of monocot mitochondrial origin. To confirm that this putative mitochondrial insertion is not the effect of an assembly artifact a PCR experiment was designed to amplify a region of approximately 2,400 bp by priming within and upstream of the insertions in both Parianinae. A second pair of primers were designed to amplify a region of similar size by priming within and downstream of the insertion in *Eremitis* sp. Note that this downstream region was not present in *P. radiciflora*. Amplification of these regions in *Eremitis* sp. produced two products that were both approximatelty 2,400 as expected. Amplification of the upstream region of *P. radiciflora* also showed a 2,400 bp product while the downstream region yielded no amplification, again as expected (Additional file 1: Figure S1). 2) A deletion of 1,500 bp unique to the represented members of the subtribe Guaduinae (*Guadua weberbaueri, Olmeca reflexa, Otatea acuminata*) is also located in approximately the same region. 3) The alignment also revealed a 150 bp inversion in the *trnD*-*psbM* intergenic spacer exclusive to all sampled members of the subtribe Olyrinae (*Cryptochloa strictiflora, Diandrolyra* sp., *Lithachne pauciflora, Olyra latifolia, Raddia brasiliensis*). 4) An insertion in the *rps16-trnQ* intergenic spacer of approximately 500 bp was located in all members of Arundinarieae sampled in this study (*Acidosasa purpurea*, *Arundinaria appalachiana*, *A. gigantea*, *A. tecta*, *Ferrocalamus rimosivaginus*, *Indocalamus longiauritus*, *Phyllostachys edulis*, *P. nigra*, *P. propinqua*, *Thamnocalamus spathiflorus*).

### Full plastome phylogenomic analysis

**Figure 2:**
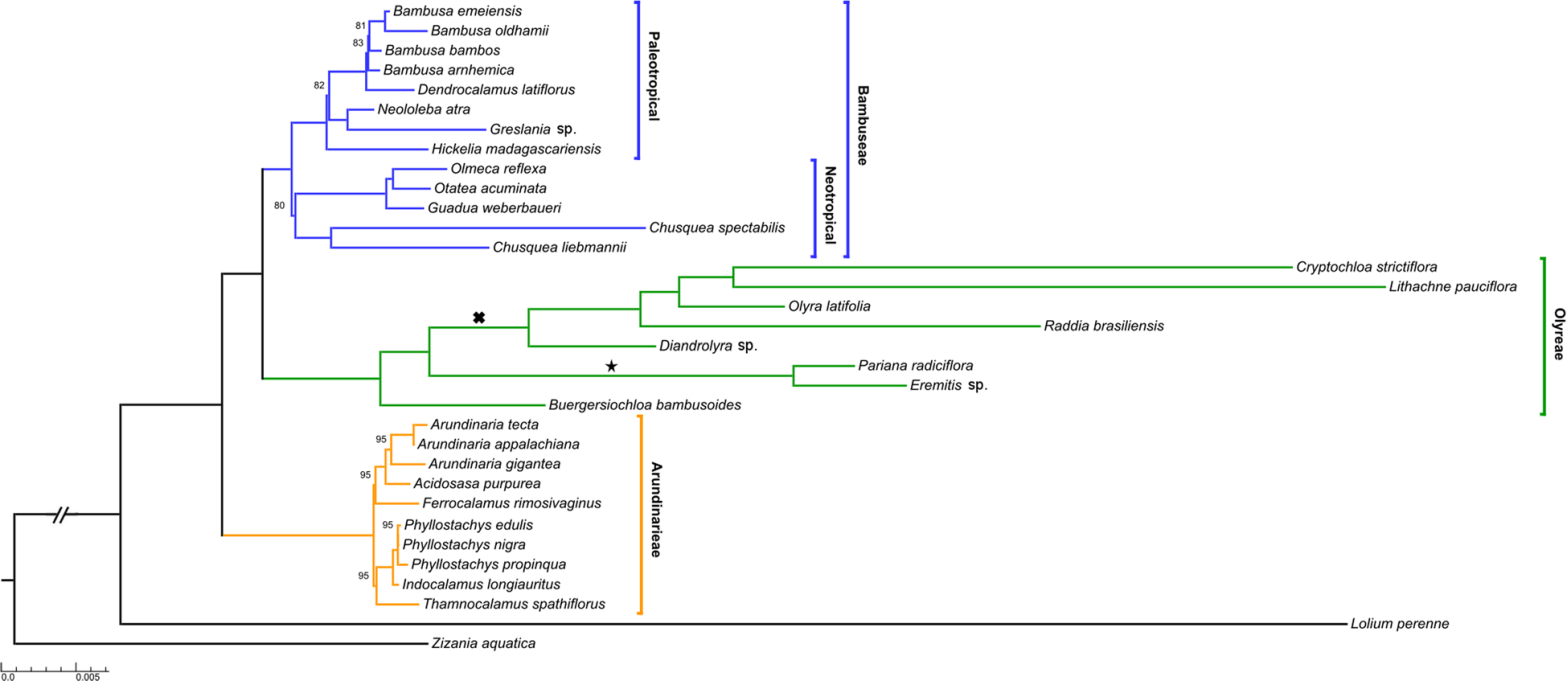
**Maximum likelihood phylogram for all complete plastomes.** Branch lengths are given in substitutions per site. The star indicates the hypothesized origin of the mitochondrion-to-plastid horizontal gene transfer event. The cross indicates the hypothesized origin of the 150 bp inversion in subtribe Olyrinae. Nodes are supported at a 100% maximum likelihood bootstrap score unless reported. All nodes were supported with a posterior probability of 1.0.

Phylogeny estimation of full plastome sequences using maximum-likelihood (ML) and Bayesian inference (BI) generated trees with identical topologies. An annotated phylogenomic tree that includes all of the taxa can be found in Figure 2. All nodes were supported in the BI analysis with a posterior probability of 1.0. These trees supported monophyly of Arundinarieae, Bambuseae, and Olyreae with Bambuseae forming a well-supported sister relationship with Olyreae. Note that this is unlikely to be an artifact of long-branch attraction because the long-branch Olyreae associate with short-branch Bambuseae rather than the long-branch outgroup taxa. The Shimodaira-Hasegawa (SH) test [24,25] allowed us to reject the alternative hypothesis of a monophyletic Bambuseae + Arundinarieae for the trees produced from these complete plastome sequences (p < 0.001). The Bambuseae diverged into two well-supported monophyletic lineages that represent neotropical and paleotropical woody bamboos. The neotropical bamboos segregated into two well-supported lineages, Chusqueinae (*Chusquea spectabilis*, *C. liebmannii*) and Guaduinae. The two representative species of Chusqueinae produced longer branch lengths than the rest of the woody bamboos with terminal branch lengths five times greater than those of the rest of Bambuseae.

The paleotropical bamboos displayed shorter branch lengths and lower support between the two sampled subtribes, Hickeliinae (*Hickelia madagascariensis*) and Bambusinae (82% ML bootstrap support). Bambusinae formed two well-supported clades: (*Dendrocalamus latiflorus* + *Bambusa* spp., and *Neololeba atra* + *Greslania* sp*.*)*.* The genus *Bambusa* was resolved as monophyletic with 83% ML bootstrap support with very short branches and one internal node with 81% ML bootstrap support (Figure 2). The Olyreae lineage demonstrated maximal support for all nodes with *Buergersiochloa bambusoides* sister to Olyrinae + Parianinae, both of which are monophyletic groups. Olyreae also displayed substantially longer branches than Bambuseae with mean internal branch lengths 5.5 times greater and mean terminal branch lengths 3.8 times greater (Figure 2). Arundinarieae was resolved into two well-supported clades: ([*Arundinaria* spp. + *Acidosasa purpurea*] + *Ferrocalamus rimosivaginus* and [*Phyllostachys* spp. + *Indocalamus longiauritus*] + *Thamnocalamus spathiflorus*). *Arundinaria* was strongly supported as monophyletic (95% ML bootstrap support) with maximum support for intrageneric relationships. *Phyllostachys* was maximally supported as monophyletic yet exhibited less intrageneric support (78% ML bootstrap value) among the three species (Figure 2).

### Phylogenetic analysis of protein-coding regions

Maximum-likelihood and Bayesian analyses of protein-coding regions showed nearly identical topologies to the full plastome analysis, including a strongly supported Bambuseae + Olyreae (Additional file 2: Figure S2). However, two differences at shallow nodes in the topology are present. The protein-coding analysis place *Dendrocalamus latiflorus* in a position embedded within the genus *Bambusa* contrasting with the sister relationship of *D. latiflorus* and *Bambusa* recovered from the full plastome analysis. *Ferrocalamus rimosivaginus* exhibits a sister relationship to the rest of the Arundinarieae, which differs from its placement sister to the *Acidosasa + Arundinaria* clade in the full plastome analysis. Additionally, seven previously recovered nodes are supported at lower ML bootstrap and posterior probabilities (see supplementary information).

## Discussion

### Plastome tree topology

The monophyletic tribes, subtribes and genera retrieved here largely confirm those identified in previous studies. Notably, the plastome tree topology demonstrates paraphyly of the entire woody bamboo syndrome and suggests two independent origins of these characters or a common origin of the syndrome followed by its loss in Olyreae. The topology within Olyreae is well-supported, which can be attributed, in part, to its higher substitution rate and increased informative sites that are likely caused by the short generation times of this annually flowering lineage [7]. The New World Chusqueinae also exhibited a higher substitution rate in our ML analyses. While some species within Chusqueinae flower as infrequently as once every 70 years, flowering intervals are extremely variable in this lineage [26-28]. *Chusquea spectabilis* was formerly classified within the genus *Neurolepis,* which has shorter flowering intervals correlating with higher altitude habitats [29]. Although the phenology of *Chusquea liebmannii* is not well known, its higher substitution rates suggest that it may also flower relatively frequently. The substitution rates of the annually flowering outgroups *Lolium perenne* [30] and *Zizania aquatica* [31] are also elevated and support the relationship between frequent reproduction and high substitution rates in the BEP clade [7].

The topology of Olyreae in our tree agrees well with current taxonomy [32]. The three recognized subtribes (Olyrinae, Parianinae, and Buergersiochloinae) are sampled here and resolved as monophyletic groups with maximum support in our phylogenomic analyses. The deep divergence of *Buergersiochloa bambusoides* is of note. Olyreae have a contemporary distribution in the New World except *Olyra latifolia* which, though largely Neotropical is also widespread in Africa/Madagascar. Another exception is *B. bambusoides*, which is endemic to New Guinea. The biogeography of Olyreae argues for a New World origin and radiation followed by limited long-distance dispersals. However, the position of *B. bambusoides* as sister to the remaining Olyreae recovered in our analysis and many others contradicts this hypothesis. Our topology suggests an Old World origin followed by a New World dispersal and radiation with a long distance dispersal event for *O. latifolia* to Africa/Madagascar, likely via birds feeding on the pseudo-berries produced by this species [29]. Other historical scenarios are more complicated invoking repeated dispersals and extinction events and are difficult to reconcile with the phylogenomic topology presented here.

Low bootstrap support and short branch lengths that obscure intrageneric relationships within *Bambusa* can be attributed to the relatively close evolutionary relationships of these species, which is reflected in high sequence similarity accompanied with a weak phylogenetic signal. Plastomes from this genus share high sequence similarity (99.8%) and fewer intrageneric synapomorphic mutations. The possibility of intrageneric hybridization as well as hybridization events between closely related genera soon after their divergence also presents an issue when the exact branching order of these species is considered [20,33]. The long generation time of the Bambuseae could allow artifacts of hybridization to persist well after their divergences.

The phylogenetic position of Greslania recovered in this study is notable (Figure 2). *Greslania* includes three or four species endemic to New Caledonia with similarities of reproductive morphology to *Hickelia* [34]. The genus is taxonomically associated with the broadly-defined Bambusinae [32], but its phylogenetic position is somewhat more specifically defined by Chokthaweepanich [35] as sister to what is called the CDMNPPS (*Cyrtochloa-Dinochloa-Mullerochloa-Neololeba-Parabambusa-Pinga-Sphaerobambos*) clade. Our well-supported phylogenomic placement of *Greslania*, sister to *Neololeba atra* (100% ML bootstrap support), is consistent with the previous work. The Australasian distribution of these taxa offers additional insights. The tectonic history of New Caledonia placed it in longstanding isolation from Australia for some 50 to 65 Ma [36]. Some endemic plants of New Caledonia are late Cretaceous relicts suggesting a distribution resulting from long-standing historical vicariance [37]. However, evidence of more recent geologic history of total submergences supports a contrasting view, that the New Caledonian flora can be no older than 37 Ma [38]. The phylogenomic position of *Greslania*, embedded within the relatively young clade of paleotropical woody bamboos, which have an estimated age ranging from 19.6 to 25 million-years [39,40] is consistent with the recent geological evidence and suggests a mid-Tertiary long-distance dispersal from a *Neololeba*-like ancestral taxon. Further complete chloroplast sampling among the CDMNPPS clade will be required to further refine the position of *Greslania*.

### Horizontal gene transfer between organellar genomes and other unique plastome features

The full plastome sequence assemblies revealed a 2,706 bp insertion of mitochondrial DNA in the *rpl23-ndhB* intergenic spacer within the IR region of *Pariana radiciflora* and a nearly identical insertion (99.6% identity) in the same region from the closely-related *Eremitis* sp., with an additional 2,232 base pairs appended to the 3′ end giving the insert a total length of 4,938 bp. A sequence similarity search using BLAST matched a 3,191 bp fragment of the insertion in *Eremitis* sp. to a region of the *rps7-atp6* intergenic spacer in the mitochondrial genome of *Ferrocalamus rimosivaginus*, a member of the Arundinarieae. Nearly the entire insert in *P. radiciflora* showed significant sequence similarity to the same region within the *F. rimosivaginus* mitochondrial genome. Although a mitochondrial fragment from Olyreae should exhibit high similarity to a mitochondrial genome from within the same lineage, only two mitochondrial genomes have been sequenced among the Bambusoideae to date (*Bambusa oldhamii* and *F. rimosivaginus*). Because of the rarity of this type of intergenomic transfer [41-43], several tests were conducted to verify accurate plastome assembly in this region. Note that this putative insertion was originally assembled de novo using Velvet (a de Bruijn graph assembler) in both taxa. The insertion was embedded in contigs of 25.9 and 42.8 kbp in *Eremitis* sp. and *P. radiciflora* respectively. Mapping the two sets of reads to their respective assemblies that include the insertion produced a continuum of perfectly overlapping reads that spanned the entire hypothesized mitochondrial insertions. The upstream and downstream boundaries of each insertion exhibited coverage of 14 and 16 respectively in *Eremitis* sp., and 124 and 107 respectively in *P. radiciflora*, which compare favorably with the overall coverage of each plastome (15.4 and 119.1, respectively). Mapping each set of reads to their respective flanking sequences produced regions identical to those of the insertion with no sign of consistent mismatching or misassembly (see Additional file 3: Figure S3). Neither were there any clear patterns of coverage interruption when approaching each locus (see Additional file 4: Figure S4). Although it could be expected that this area would show higher coverage due to the reference mapping using reads from both the plastid and mitochondrial regions, the inserts show comparable coverage to the rest of the genome. The mitochondrial inserts are represented sufficiently to produce an assembly but are not proportionally represented in the read pool. One possible explanation could be that the base composition in the mitochondrial inserts is less AT rich than in the rest of the plastome (55% compared to 61%) as the PCR step during Illumina library preparation has been shown to introduce base composition bias in fragments [44]. Another possibility is that this region was deleted from the mitogenome in the course of transfer to the plastome.

It should also be noted that the Illumina libraries for *P. radiciflora* and *Eremitis* sp. were prepared using different methods (TruSeq Nano and Nextera respectively), sequenced at different facilities using paired-end and single-end, respectively, and plastomes were assembled independently using completely *de novo* methods. Finally, the insert was confirmed with a PCR experiment using plastome/mitogenome primer pairs.

We hypothesize that this event originated from a recombination between the plastome and the homologous regions within the mitochondrial genome most probably in a common ancestor of these two taxa. The appearance of this mitochondrial insertion in two species of Parianinae is striking, and most parsimoniously interpreted as a single event even though one of the inserts is 2.2 kbp longer than the other. Given the rarity of mitochondrial insertions in grass plastomes, two such similar events in closely related taxa is more difficult to explain than a single insertion with subsequent differential degradation of this noncoding DNA. Events in which a mitochondrial genome incorporates DNA sequences of plastome origin are not rare, especially in bamboos [45]. This creates homologous regions between the cytoplasmic organelles, which following further mitochondrial rearrangements might facilitate recombination of additional mitochondrial sequences into the chloroplast. While the mitochondrial genomes have yet to be sequenced in *P. radiciflora* and *Eremitis* sp., querying the mitochondrial genome of *Bambusa oldhamii* with its own plastome sequence using BLASTn reveals over forty regions of significant sequence similarity longer than 100 bp in length. The much less frequent horizontal gene transfer from mitochondrion to plastid has been observed and verified in other plant species [41-43].

The subset of Olyreae that possess the unique 150 bp inversion in the *trnD*-*psbM* intergenic spacer includes representatives of only five genera of Olyrinae. The Olyrinae are well-supported as monophyletic in this study as is also suggested in Oliveira et al. [46], which indicates that this inversion likely occurred once in the common ancestor of this lineage. The high number of substitutions and indels accumulated between species within this unique inversion either supports the notion that the inversion event occurred early in the history of this lineage or that mutation rates are elevated. An imperfect eight bp inverted repeat flanking the inverted region (CCYTTTTY -inversion- GAAAAAGG) suggests that a possible inversion mechanism could be a stem-loop formation induced recombination.

## Conclusions

This study successfully characterizes the full plastome sequences of 16 tropical bamboos, one temperate bamboo, and one ehrhartoid grass. Three sequences from Guaduinae and two from Chusqueinae represent the first completely assembled plastome sequences from the New World tropical woody lineage. Though full plastome sequences have been assembled from Old World tropical species [9,16] our taxonomic sampling of this lineage extends beyond the *Bambusa* - *Dendrocalamus* clade. This study also marks the first full plastome phylogenomic analysis to be performed within Olyreae. Two plastomes from Olyreae reveal the first evidence of a synapomorphic mitochondrial-to-plastid horizontal gene transfer in monocots.

**Figure 3:**
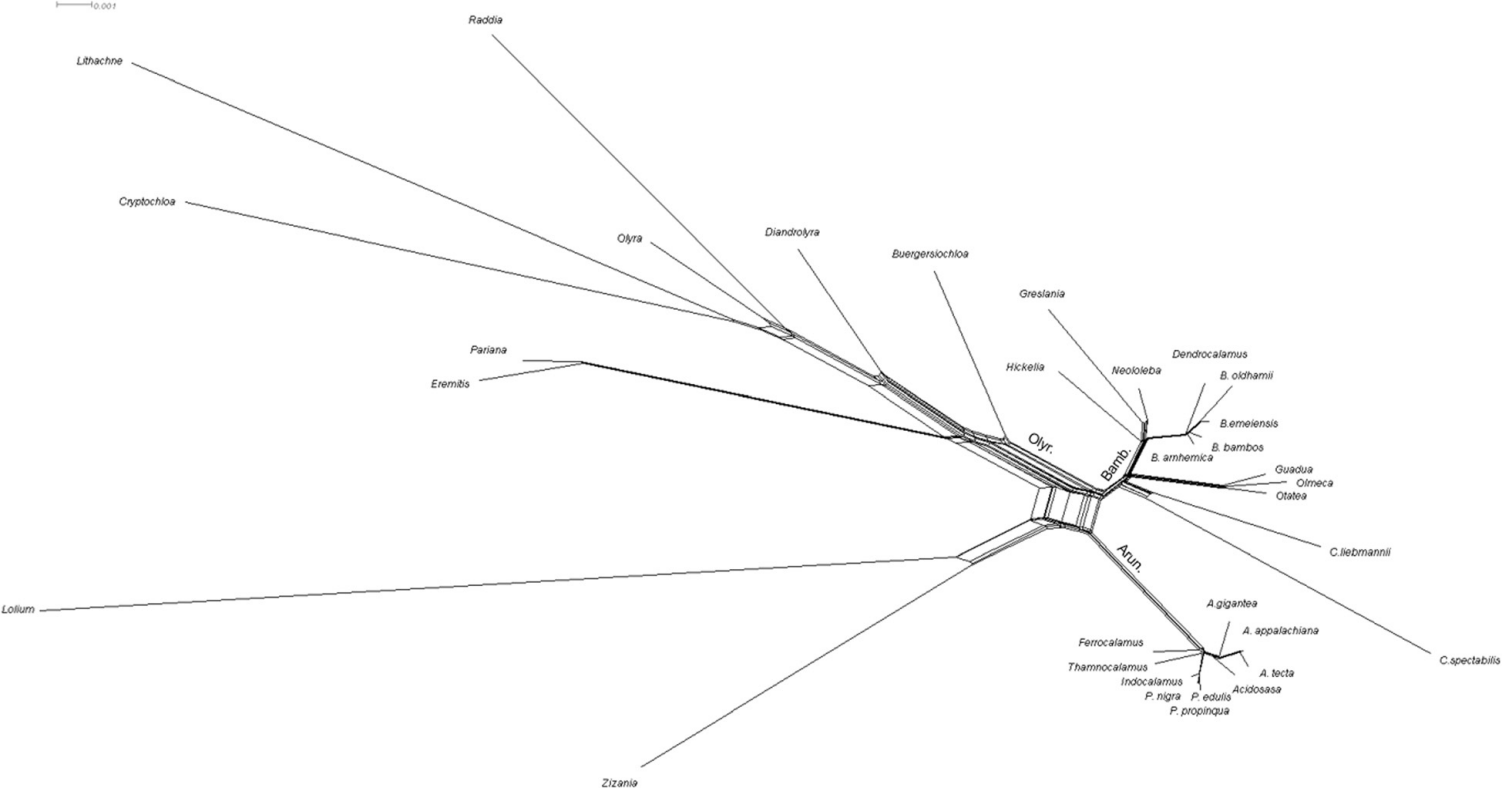
**A neighbor-net analysis indicates conflicting phylogenetic signals in the data.** The three main bamboo lineages are indicated. Note the branches for outgroup taxa *Lolium* and *Zizania* were truncated to facilitate visibility. Bamb.: Bambuseae; Olyr.: Olyreae; Arun.: Arundinarieae.

This phylogenomic study supports paraphyly of the woody bamboo syndrome. However, the scope of the relationships presented here is restricted to the maternally inherited evolutionary signal, which demonstrates considerable conflict in the phylogenomic network analysis at the node where the three main lineages diverge (Figure 3). A study on three single-copy nuclear markers performed by Triplett et al. [15] outlined a scenario in which the extant allopolyploid woody bamboos are a result of two separate hybridization events between at least four distinct precursor lineages. Herbaceous bamboos were supported in a sister relationship to a progenitor lineage that eventually diversified into precursor lineages that hybridized to form the extant woody bamboos. The Triplett et al. [15] study clarified some of the complexities of bamboo diversification and provided evidence that the apparent paraphyly of the woody syndrome in bamboos may be an artifact of analysis with exclusively plastid loci. However, note that one out of the three nuclear markers potentially supported the robust tropical woody-herbaceous bamboo sister relationship in plastid studies by embedding the diploid herbaceous clade within lineages exclusive to tropical woody bamboos. Further study using a wider variety of nuclear markers may clarify this significant event in bamboo diversification.

Additionally, a comparative study on the transcriptomics of the lignin biosynthesis and deposition pathways could provide further insight on the evolution of the woody character. A single origin of characters found in the woody bamboo syndrome would be supported by similar expression profiles between Bambuseae and Arundinarieae in the genes for enzymes and transcription factors involved in lignin biosynthesis and deposition and formation of bisexual florets while differing expression profiles could suggest otherwise. Other potential expansions on this study are an examination of the phylogenetic signals given by other molecular characters such as mitochondrial coding sequences and microstructural changes.

## Methods

### Taxon sampling and DNA extraction

Silica-dried leaf tissue was obtained from 17 species of bamboos (*Bambusa arnhemica* F.Muell., *Bambusa bambos* (L.) Voss, *Buergersiochloa bambusoides* Pilg., *Chusquea liebmannii* E. Fourn. ex Hemsl., *Chusquea spectabilis* L.G. Clark., *Diandrolyra* Stapf. sp., *Eremitis* Döll sp., *Greslania* Balansa sp., *Guadua weberbaueri* Pilg., *Hickelia madagascariensis* A. Camus, *Lithachne pauciflora* P. Beauv., *Neololeba atra* (Lindl.) Widjaja, *Olmeca reflexa* Soderstr., *Otatea acuminata* (Munro) C.E. Calderón & Soderstr., *Pariana radiciflora* Sagot ex Döll, *Raddia brasiliensis* Bertol., and *Thamnocalamus spathiflorus* Munro) and one ehrhartoid species (*Zizania aquatica* L.). Herbarium voucher specimens were collected and are reported in Table 1. Tissue was homogenized manually in liquid nitrogen before extraction. The DNA extraction protocol using the Qiagen DNeasy Plant Mini Kit (Qiagen Inc., Valencia, CA) was followed.

### Illumina sequencing and quality control

Starting quantities of total genomic DNA from *Bambusa arnhemica*, *B. bambos*, and *Thamnocalamus spathiflorus* were determined by measurement at A260 with a Nanodrop 1000 (ThermoFisher Scientific, Wilmington, DE, USA) to be approximately 1.5 μg each. DNAs were diluted to approximately 2 ng/μl and sheared into ~300 bp fragments using a Bioruptor® sonicator (Diagenode, Denville, NJ, USA) in two 12 min., periods, inverting the tubes between periods. Sonicated DNA preparations were purified and concentrated with the MinElute Gel Extraction Kit (Qiagen Inc., Valencia, CA, USA). Single read libraries were prepared using the TruSeq sample preparation low throughput protocol (gel method) following manufacturer instructions (Illumina, San Diego, CA, USA). Sequencing was performed on a HiSeq 2000 instrument (Illumina, San Diego, CA, USA) using single reads at the Iowa State University DNA Sequencing Facility, Ames, IA, USA. Reads produced by this method were 99 bp in length.

Quantities of total genomic DNA from *Chusquea liebmannii*, *Otatea acuminata* and *Pariana radiciflora* were determined using the Qubit fluorometric quantitation system (Life Technologies, Grand Island, NY, USA). Two micrograms were used in each library preparation. Libraries were prepared using the TruSeq Nano DNA sample preparation kit (Illumina, San Diego, CA, USA) and sequenced paired-end at Cold Spring Harbor laboratory, Cold Springs, New York, USA.

Total genomic DNA extracts for the remaining taxa were diluted to 2.5 ng/ul in 20 ul water. The Nextera Illumina library preparation kit (Illumina, San Diego, CA, USA) was used to prepare libraries for sequencing and the DNA Clean and Concentrator kit (Zymo Research, Irvine, CA, USA) was used for library sample purification. Sequencing was performed with the HiSeq 2000 instrument at the Iowa State University DNA Sequencing Facility, Ames, USA using single reads. This method produced 100 bp fragments. See Table 2 for details on sequencing techniques for each respective taxon.

All reads were first quality filtered using DynamicTrim v2.1 from the SolexaQA software package [47] with default settings, and then sequences less than 25 bp in length (default setting) were removed with LengthSort v2.1 in the same package.

### Plastome assembly, annotation, and alignment

Plastome assembly was performed with entirely de novo methods. The Velvet software package [48] was run iteratively by loading previously assembled contigs into the Velvet assembler multiple times (see Wysocki et al. [49] for details) with kmer lengths ranging from 19–85 bp increasing by steps of 6 bp. Contigs were scaffolded using the anchored conserved region extension (ACRE) method [49]. Because Velvet and other de Bruijn graph-based programs cannot build across repeated areas (such as the inverted repeat regions in angiosperm plastomes), the plastome is assembled in segments, which need to be manually joined. The number of contigs scaffolded for each taxon is reported in Table 2. Any remaining gaps in the plastomes were resolved using contigs or reads by locating overlapping regions of at least 20 bp that had zero mismatches and started at one end of the read or contigs. Paired-end reads that were used to resolve gaps were verified by checking the position and orientation of their downstream mate. Gaps were resolved until the circular map was complete with no gaps or ambiguities. Overlapping regions were identified and gap closure was performed using Geneious Pro (Biomatters Ltd., Auckland, NZ). A final assembly assessment was performed by mapping each set of reads to their respective plastome and locating any sequence inconsistencies as described in Wysocki et al. [49]. Assembled plastomes were annotated by aligning to a closely-related and previously annotated reference plastome in Geneious Pro and transferring the annotations from the reference to the assembled plastome when the annotation shared a minimum similarity of 70%. The banked plastomes from *Arundinaria gigantea* (NC020341), *Bambusa oldhamii* (NC012927) and *Cryptochloa strictiflora* (JX235348) were used as annotation references for members of the tribes Arundinarieae, Bambuseae and Olyreae respectively.

A PCR experiment was performed to verify putative mitochondrial insertions in the *Pariana radiciflora* and *Eremitis* sp. plastomes. Two pairs of primers were used to amplify fragments in which mitochondrial sequence was found adjacent to plastid sequence. Two primers were designed based on the mitochondrial insert sequence in *Eremitis* sp. A BLASTn search of these two primer sequences showed 96-100% nucleotide identity to the *Ferrocalamus rimosivaginus* mitochondrial genome and no significant similarity to banked chloroplast sequences. The other pair of primers were chosen based on flanking chloroplast sequence. Each pair of primers included one that annealed inside the insertion and one that annealed outside. The amplified fragments spanned insertion termini. The three designed primers were: 5′-GGGTCTCATCTGAAGGGAGGCAGGC-3′, 5′-GTGAGGCAGGTTCTCATGGTTCGG-3′ and 5′-GTGCTATCGGATCGGGTGAATTAGAG-3′, and the IRb 3 F primer from Dhingra and Folta [50] was also used. Amplifications were performed using the Fidelitaq system (Affymetrix, Santa Clara, CA) following the manufacturer’s protocol. Products were separated electrophoretically on an agarose gel system (Additional file 1: Figure S1).

Plastomes were arranged, beginning at the 5′ end, with the large single copy region (LSC) followed by the inverted repeat region B (IRb), ending with the short single copy region (SSC). Inverted repeat region A was omitted from the matrix to be used for phylogenomic analysis to prevent overrepresentation of the inverted repeat sequence. The assembled plastomes were then aligned, along with 14 previously published bambusoid plastomes and one pooid and one ehrhartoid grass plastome each, using the MAFFT alignment software [51]. The alignment was then inspected for structural mutations and adjusted manually to preserve tandem repeat boundaries and identify inversions. Regions that contained inversion mutations were deleted to remove false homology inferences. To test for potential differences in phylogenetic signal, all protein coding sequences were extracted from the alignment and concatenated for partitioned analyses.

### Phylogeny estimation

Nucleotide positions that contained one or more gaps introduced by the alignments were omitted from the matrix. The Akaike Information Criterion (AIC) was used in the jModelTest software package v 2.1.3 [52,53] to compare models of character evolution. The General Time Reversible model of substitution, incorporating invariant sites and a gamma distribution (GTR + I + G), was among a group of equally best fit models (found in the 100% confidence interval) and was used in subsequent plastome analyses. Maximum likelihood analysis was performed using the RAxML v 8.0.5 software package [54] with 1,000 non-parametric bootstrap replicates. Outgroup choice for Bambusoideae is complicated by the fact that divergence and radiation of the BEP subfamilies, possibly combined with undocumented extinctions, puts any candidate outgroup for Bambusoideae on comparatively long branches in phylogenetic trees [17]. This creates the potential for introducing phylogenetic artifacts. Full plastomes from the ehrhartoid grass *Zizania aquatica* (this paper) and the pooid grass *Lolium perenne* (NC009950) were included in the matrix as outgroup taxa. Non-parametric bootstrap values were generated using the Consense function of the Phylip software package [55]. An alternate topology was tested for the complete plastome partition in the likelihood framework. A second ML analysis was performed constraining the woody species to be monophyletic specifying identical parameters in the RAxML software. Constrained and unconstrained analyses were compared using the Shimodaira-Hasegawa (SH) test function included in PAUP* [56]. MrBayes 3.2.2 [57] was used to perform a Bayesian inference analysis. The Markov chain Monte Carlo (MCMC) analysis was run for 2 X 10,000,000 generations. Average standard deviation of split frequencies remained below 0.001 after the fifty percent burn-in. A neighbor-net analysis was then performed on the full plastome alignment to visualize character state conflict using the SplitsTree4 v. 4.13.1 [58].

### Availability of supporting data

The data set supporting the results of this article is available in Dryad and can be found at http://datadryad.org/resource/doi:10.5061/dryad.7qc22 [59]. Data were also deposited in the TreeBASE repository, http://purl.org/phylo/treebase/phylows/study/TB2:S16364. All nucleotide sequences were deposited in the NCBI Genbank repository. Accessions can be found in Table 1.

## Additional files

Additional file 1: Figure S1. Photograph of an agarose gel showing PCR products used to verify the presence of mitochondrial inserts in the *Eremitis* sp. and *Pariana radiciflora* plastomes. A modified version of Figure 1 is displayed to show the position of each product. Primer annealing sites are indicated with arrows and expected amplification products are represented with bars. Black bars indicate products that amplified and gray bars indicate areas where no priming would be expected to produce a product. Each reaction is labeled A through F as products in the diagram and lanes in the photograph. The N indicates the negative control reaction containing no template DNA. Note that this figure is not drawn to scale. Additional file 2: Figure S2. Maximum likelihood consensus cladogram for concatenated analysis of protein coding sequences. Nodes are supported at a 100% maximum likelihood bootstrap score unless reported (first value). Nodes were supported with a posterior probability of 1.0 unless reported (second value). Additional file 3: Figure S3. The unfiltered read set from *Eremitis* sp. mapped to the region flanking the 5′ and 3′ borders of the ~5 kbp insertion in the inverted repeat of the plastome. Regions corresponding to typical chloroplast sequence and the mitochondrial insertion are indicated. Mismatches to the consensus sequence are indicated with color. Reference mapping and visualizations were performed with Geneious Pro v 7.1.2. The regions flanking the insertion in the *Pariana radiciflora* plastome have higher levels of coverage (not shown). Additional file 4: Figure S4. Two graphs indicating the coverage of each nucleotide position in the *Eremitis* sp. and *Pariana radiciflora* plastomes. The position of the mitochondrial insertion in each plastome is shown with red bars indicating the start and end of each. Note that only one inverted repeat is shown here.

## Abbreviations

BEP: Bambusoideae- Ehrhartoideae-Pooideae
bp: Base pairs
IR: Inverted repeat
indel: Insertion/ deletion
ML: Maximum-likelihood
BI: Bayesian inference
LRT: Likelihood ratio test
CDMNPPS: *Cyrtochloa*-*Dinochloa*-*Mullerochloa*-*Neololeba*-*Parabambusa*-*Pinga*-*Sphaerobambos*
ACRE: Anchored conserved region extension
LSC: Large single-copy
SSC: Short single-copy
AIC: Akaike information criterion
GTR: General time reversible
MCMC: Markov chain Monte Carlo
