# Supplementary figures and images for: Evolution of the bamboos (Bambusoideae; Poaceae): a full plastome phylogenomic analysis
Source: BMC Evol Biol. 2015 Mar 18;15:50. doi: 10.1186/s12862-015-0321-5 (PMC4389303; doi:10.1186/s12862-015-0321-5)

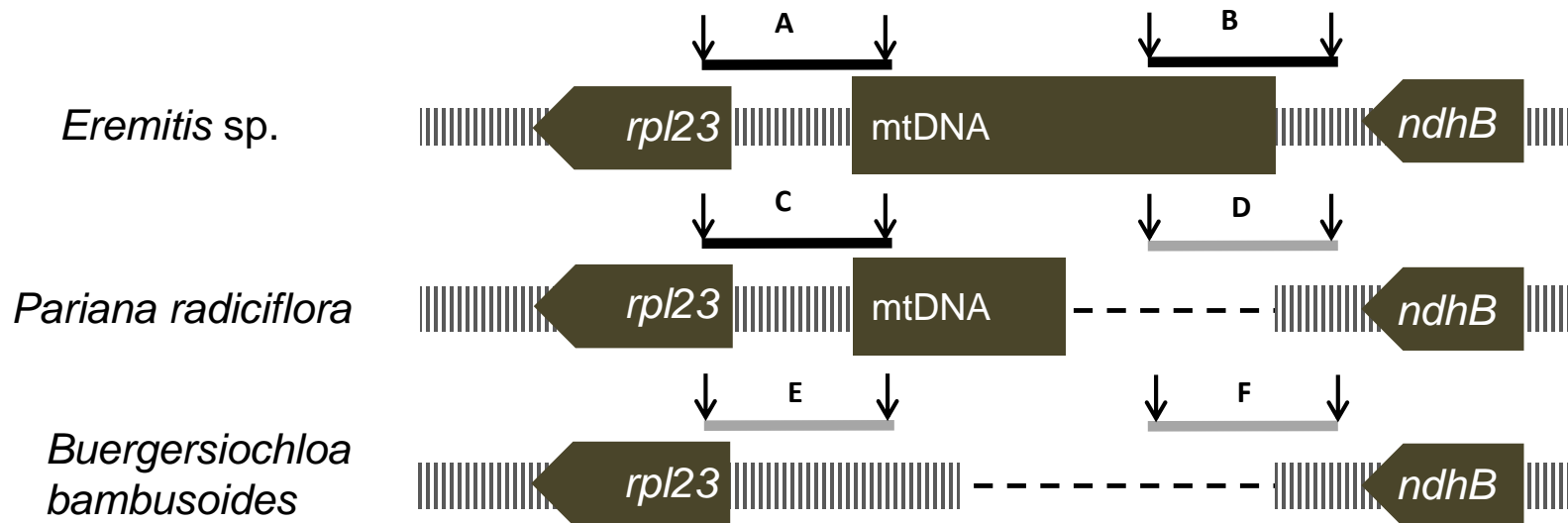

Ladder  
(bp)

A B C D E F N

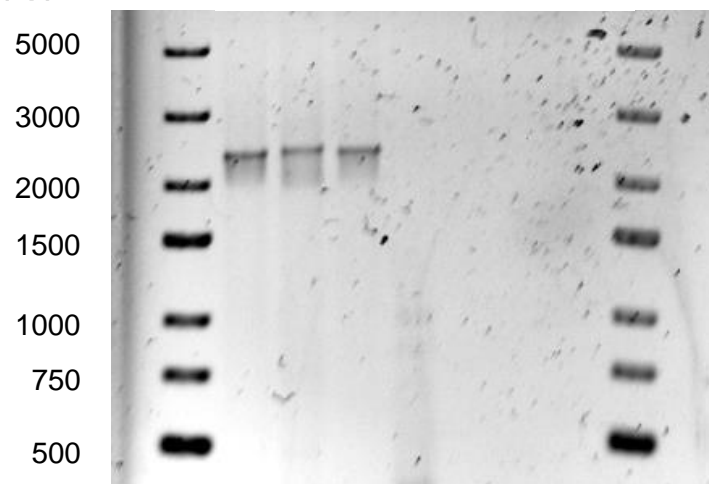

Supplement: Additional file 1: Figure S1. — Photograph of an agarose gel showing PCR products used to verify the presence of mitochondrial inserts in the Eremitis sp. and Pariana radiciflora plastomes. A modified version of Figure 1 is displayed to show the position of each product. Primer annealing sites are indicated with arrows and expected amplification products are represented with bars. Black bars indicate products that amplified and gray bars indicate areas where no priming would be expected to produce a product. Each reaction is labeled A through F as products in the diagram and lanes in the photograph. The N indicates the negative control reaction containing no template DNA. Note that this figure is not drawn to scale. [file 12862_2015_321_MOESM1_ESM.pdf]

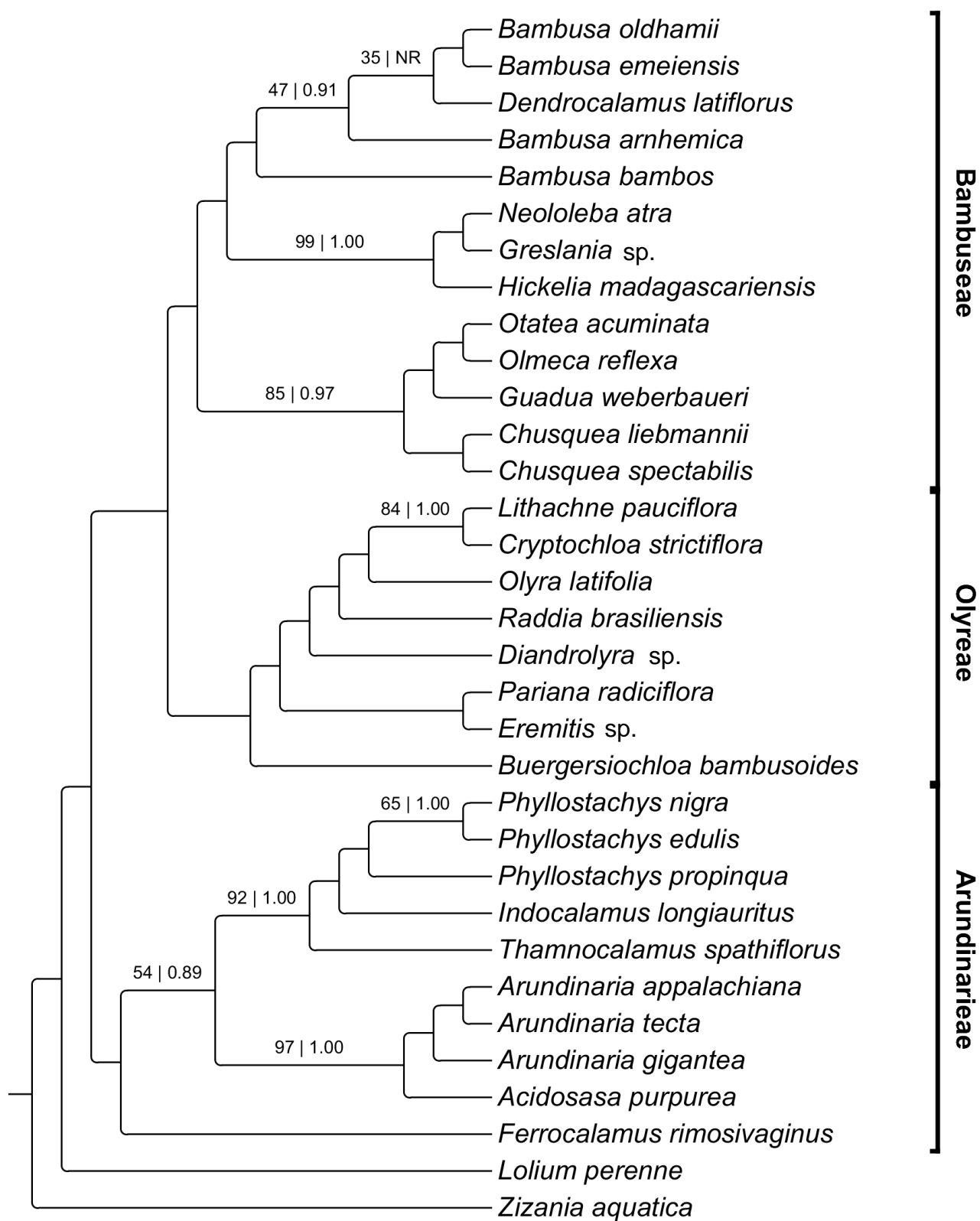

Supplement: Additional file 2: Figure S2. — Maximum likelihood consensus cladogram for concatenated analysis of protein coding sequences. Nodes are supported at a 100% maximum likelihood bootstrap score unless reported (first value). Nodes were supported with a posterior probability of 1.0 unless reported (second value). [file 12862_2015_321_MOESM2_ESM.pdf]

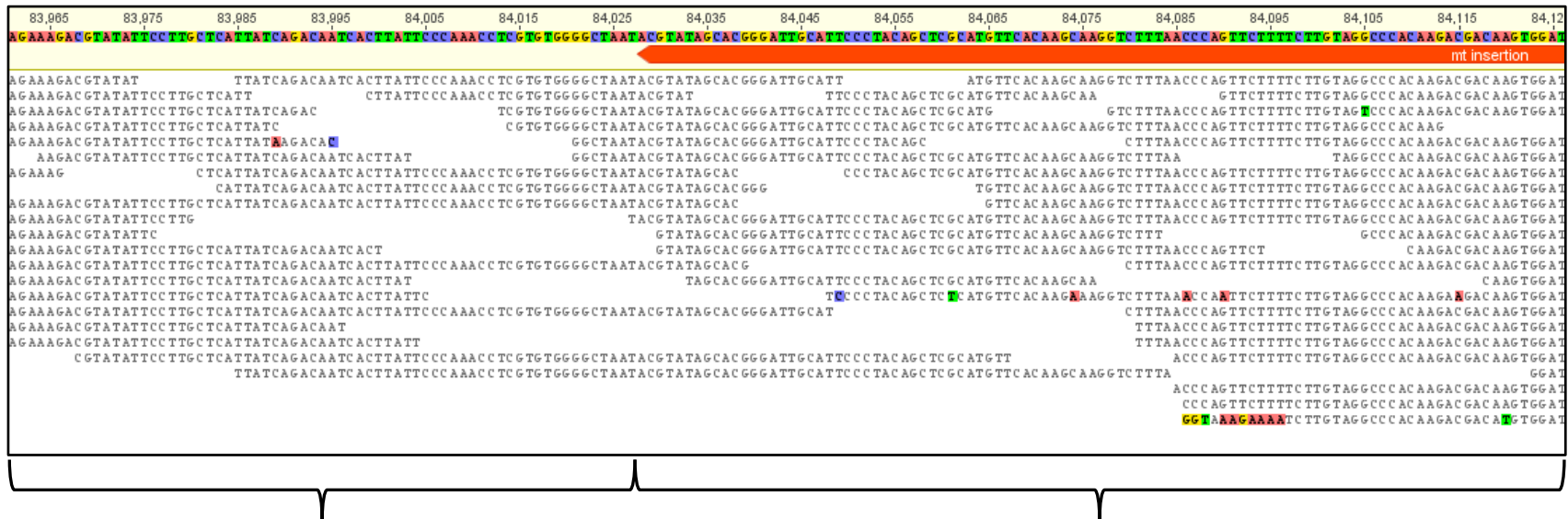

Chloroplast

Mitochondrial

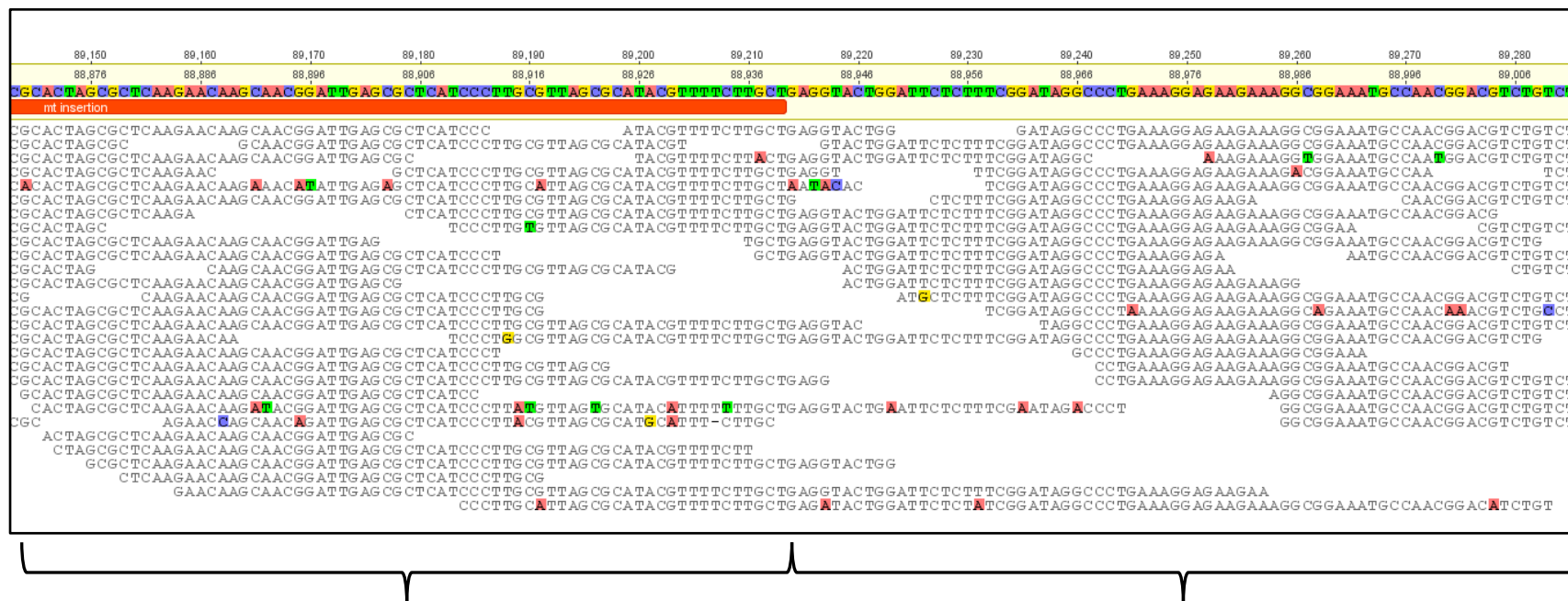

Mitochondrial

Chloroplast

Supplement: Additional file 3: Figure S3. — The unfiltered read set from Eremitis sp. mapped to the region flanking the 5′ and 3′ borders of the ~5 kbp insertion in the inverted repeat of the plastome. Regions corresponding to typical chloroplast sequence and the mitochondrial insertion are indicated. Mismatches to the consensus sequence are indicated with color. Reference mapping and visualizations were performed with Geneious Pro v 7.1.2. The regions flanking the insertion in the Pariana radiciflora plastome have higher levels of coverage (not shown). [file 12862_2015_321_MOESM3_ESM.pdf]

***Eremitis* sp.**

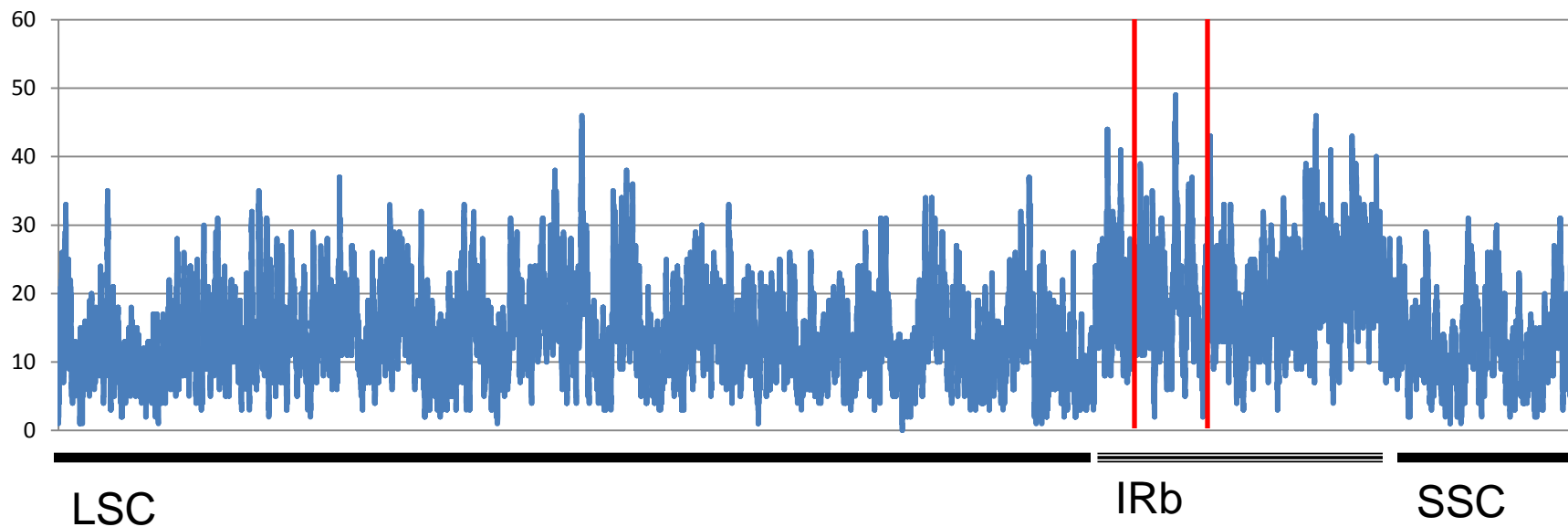

***Pariana radiciflora***

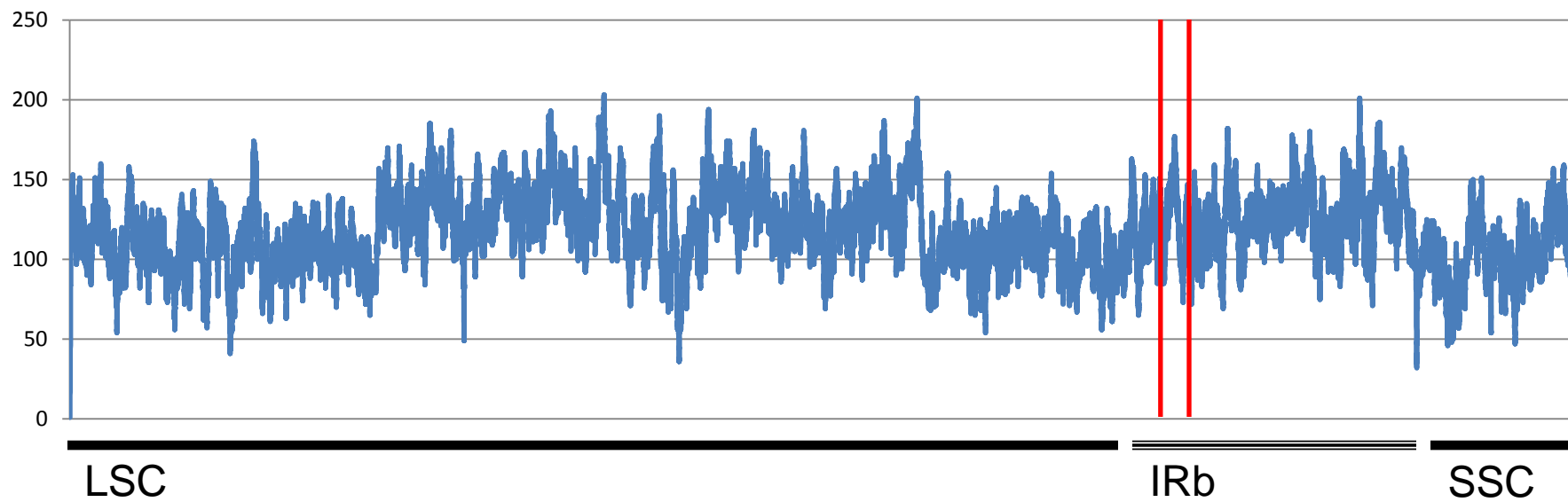

Supplement: Additional file 4: Figure S4. — Two graphs indicating the coverage of each nucleotide position in the Eremitis sp. and Pariana radiciflora plastomes. The position of the mitochondrial insertion in each plastome is shown with red bars indicating the start and end of each. Note that only one inverted repeat is shown here. [file 12862_2015_321_MOESM4_ESM.pdf]
